# Reducing the Crystallite Size of Spherulites in PEO-Based Polymer Nanocomposites Mediated by Carbon Nanodots and Ag Nanoparticles

**DOI:** 10.3390/nano9060874

**Published:** 2019-06-09

**Authors:** Ranjdar M. Abdullah, Shujahadeen B. Aziz, Soran M. Mamand, Aso Q. Hassan, Sarkawt A. Hussein, M. F. Z. Kadir

**Affiliations:** 1 Hameeds Advanced Polymeric Materials Research Laboratory, Department of Physics, College of Science, University of Sulaimani, Qlyasan Street, Sulaimani 46001, Kurdistan Regional Government, Iraq; ranjdar.abdullah@univsul.edu.iq (R.M.A.); soran.mamand@univsul.edu.iq (S.M.M.); sarkawt.hussen@univsul.edu.iq (S.A.H.); 2 Komar Research Center (KRC), Komar University of Science and Technology, Sulaimani 46001, Kurdistan Regional Government, Iraq; 3 Department of Chemistry, College of Science, University of Sulaimani, Qlyasan Street, Sulaimani 46001, Kurdistan Regional Government, Iraq; asoqadir2012@yahoo.com; 4 Centre for Foundation Studies in Science, University of Malaya, Kuala Lumpur 50603, Malaysia; mfzkadir@um.edu.my

**Keywords:** PEO nanocomposite, carbon nano dots, plasmonic silver nanoparticles, XRD study, morphology, optical properties

## Abstract

The PEO-based polymer nanocomposites were prepared by solution cast method. Green approaches were used for synthesis of carbon nanodots (CNDs) and silver nanoparticles (Ag NPs). It was found that the crystallite size of spherulites of PEO was greatly scarified upon incorporation of CNDs and Ag NPs. In the present work, in opposition to other studies, broadening of surface plasmon resonance (SPR) peak of metallic Ag NPs in PEO-based polymer composites was observed rather than peak tuning. Various techniques, such as powder X-ray diffraction (XRD), SEM, UV–Vis spectroscopy, and photoluminescence (PL), were used to characterize the structural, morphological, and optical properties of the samples. Increase of amorphous phase for the PEO doped with CND particles was shown from the results of XRD analyses. Upon the addition of suspended Ag NPs to the PEO:CNDs composites, significant change of XRD peak position was seen. A field-emission scanning electron microscope (FESEM) was used to investigate the surface morphology of the samples. In the SEM, a significant change in the crystalline structure was seen. The size of PEO spherulites in the PEO nanocomposite samples became smaller and the percentage of amorphous portion became larger, owing to the distribution of CNDs and Ag NPs. The UV–Vis absorption spectra of the PEO-based polymer were found to improve and shift to higher wavelengths upon incorporation of CNDs and Ag NPs into the PEO matrix. The SPR peak broadening in the UV–Vis spectra was observed in the PEO:CNDs composites due to the Ag NPs. The absorption edge value of PEO was found to shift toward lower photon energy as the CNDs and Ag NPs are introduced. The photoluminescence (PL) spectra were also observed for the PEO:CNDs and PEO:CNDs:Ag samples and found to be more intense in the PEO:CNDs system than in the PEO:CNDs:Ag system. Lastly, the optical band gap of the samples was further studied in detail using of Tauc’s model and optical dielectric loss parameter. The types of electron transition were specified.

## 1. Introduction

Recent studies have reveal that optical properties of polymer composites have attracted great attention of many researchers due to their wide application in various fields, such as solar cell, light-emitting diodes (LEDs), and optoelectronic devices [1,2,3,4,5,6,7]. Adding nano-size particles into polymers is known to be crucial for photonic and optoelectronic applications [8,9,10,11,12]. Polymers are generally considered to be an excellent host material for inorganic nanoparticles (NPs). However, poly(ethylene oxide) (PEO) as a polymer matrix has been shown to exhibit inherent problems when incorporates with alkali metal salts [13]. PEO is relatively inexpensive and water soluble material. It is a linear and semicrystalline polymer with a helical structure below melting point (60 °C) [14]. An intensive and extensive study of literature revealed that very limited works have been carried out on optical properties of PEO-based polymer [15]. Due to the high-surface to bulk ratio of the inorganic NPs, their incorporation into the host polymer can lead to a drastic change in the properties of the host [16]. The rapid advancement of nanotechnology in recent years has led to develop new and more effective methods to prepare NPs. So far, various physical and chemical methods have been adopted, such as microwave and UV irradiations, chemical reduction [17], laser ablation, chemical/physical vapor deposition, pyrolysis, sol gel [18] and etc. However, due to the presence of toxicity, hazardous chemical materials, difficulty in purification, low material conversions, and demand for high energy in these preparation methods, green synthesized method employing either biological microorganisms or plant extracts has been used instead, as a more environmentally friendly option [17,18,19,20,21]. The type of plant extract as it is used in our work has been considered to be more convenient and powerful compared with the biological microorganisms type [17]. It is well reported that green processes are promising methods for the synthesis of nanoparticles and their outcomes are found to be nontoxic and environmentally friendly [19]. The most popular plants used for the preparation of NPs were found to be Camellia sinensis [17,20,22,23,24], Arbutus Unedo, Ocimum [25], Rosa rugosa [26] and etc. In the field of nano-science, silver (Ag) NPs are well known of having numerous applications in our daily life, owing to their excellent physical and chemical properties, such as high thermal and electrical conductivities and catalytic effect [27]. Consequently, they can also possess valuable medical and biological properties, such as antibacterial, anti-inflammatory, antifungal, and antiviral properties [17,28]. The progress of Ag NPs is expanding in recent years. They are currently used as wound dressings, component of clothes, embed coat, food container, and balms [17]. Noble metal NPs can also exhibit unique optical properties, such as scattering of light and resonant absorption, different from their bulk counterparts. This is due to the existence of collective coherent excitations of free electrons in their conduction band [29]. On the other hand, the invention of carbon nanodots (CNDs) and carbon-based nanomaterials has been commonly investigated. Currently, CNDs represent a newest class of carbon-based materials for sustainable applications. It is considered as a potential alternative to carbon nanotubes (CNTs) [30]. The CNDs are discrete nanoparticles with quasi-spherical shape that are ultrafine (≤10 nm) in size. They can be used as building block components of fluorescence systems [2,31]. Several beneficial features of CNDs, such as fluorescence behavior, biodegradability, abundance of carbon sources, and low-cost, have made them to be widely used in many applications, such as drug delivery, biological and biomedical imaging, energy, photovoltaic devices, and optoelectronics [31,32]. In addition, CNDs have other important benefits over conventional fluorescent organic dyes and semiconductor quantum dots, owing to their relatively high resistivity to photobleaching, inertness, and chemical stability in the colloidal solution state [31]. In the current study, the influence of CNDs and Ag NPs on crystallite size of spherulites of PEO was examined through the field-emission scanning electron microscope (FESEM) technique. The influence of CNDs and Ag NPs on UV absorption and their effects on optical properties of PEO-based solid polymer composites are also investigated, tending to enhance of absorption of ultraviolet (UV) radiation, which is important for shielding application.

## 2. Experimental Methodology

### 2.1. Composite Preparation

In this study, PEO powder with molecular weight of 5 × 10^6^ g/mole purchased from Sigma Aldrich was used. For the preparation of PEO-based composites, the solution cast technique was adopted. Three batches of 1 g of PEO dissolved in 50 mL of acetonitrile were prepared. A homogeneous solution of CNDs was obtained by adding 45 mL of distilled water to the CNDs (5 mg) with continuous stirring. A hydrothermal treatment of glucose was performed to obtain a bright yellow CND solution as follows; first, 1 g of glucose was added to a 5 mL of concentrated phosphoric acid to make a colorless solution. Then, the solution was heated in a water bath between 80 °C and 90 °C for 20 to 30 min until a dark brown solution forms. Next, the solution was cooled down to room temperature and diluted NaOH added to adjust the pH between 3 and 4, afterwards, it was left overnight. The purification of CNDs was carried out using chloroform and then evaporation of the chloroform was performed. More details of CNDs preparation can be seen in our earlier work [2]. To prepare PEO-based nanocomposites; the suspended CNDs (10 mL) was added to one of the dissolved PEO solution. On the other hand, Ag NP fabrication was undertaken through the green method. Quince leaves were utilized to obtain natural colorants, which are enriched with phenolic compounds. It is situated in the medium of conjugated double bonds where silver ions are reduced to metallic NPs. For the purposes of this study, unfermented quince leaves were used. The green leaves acquired from the quince trees were washed with distilled water. After drying at room temperature, the leaves were protected from sun exposure for 7 days. The natural colorant of the leaves was then extracted. The detail of synthesis of Ag NPs using quince leave extract can be seen in our previous article [33]. The obtained solution of Ag NPs at ambient temperature was then centrifuged to obtain the deposit of Ag NPs. Subsequently, the precipitated Ag NPs were repeatedly washed with distilled water. The Ag NPs were dispersed in 50 mL distilled water. Furthermore, 10 mL of suspended Ag NPs was finally added to the PEO solution mediated by 10 mL CNDs to obtain PEO:CNDs:Ag. The three samples were labelled as PEPN0, PEPN1 and PEPN2 for pure PEO, PEO:CNDs, and PEO:CNDs:Ag, respectively. The obtained samples were stirred for 3 h by using a magnetic stirrer. It was then poured into a sterilized Petri dishes and vacuum-dried at ambient temperature. Finally, the samples were further dried in glass desiccators.

### 2.2. Characterization Techniques

The samples were analysed using powder X-ray diffraction (XRD), field-emission scanning electron microscope (FESEM), ultraviolet–visible (UV–Vis) spectroscopy, and FTIR spectroscopy. X-ray diffraction patterns were recorded on an Empyrean XRD (PANalytical, Netherland) with the operating current and voltage of 40 mA and 40 kV, respectively. The samples were scanned with a beam of monochromatic CuKα X-radiation (λ = 1.5406 Å) in a glancing angle range of 5° ≤ 2θ ≤ 80° with step size of 0.1°. To analyse the surface microstructure of the nanocomposites, field-emission scanning electron microscope (FESEM) images, at adjusted magnification, were captured using Hitachi SU8220 FESEM with 100× magnification. The (UV–Vis) spectra of the samples were detected on V-570 UV–Vis spectrometer (Jasco, Japan) with a scanning range of 180 to 1000 nm. Fluorescence spectra were acquired using a Cary Eclipse fluorescence spectrophotometer (Agilent, Santa-Clara, CA, USA).

## 3. Results and Discussion

### 3.1. XRD Study

**Figure 1:**
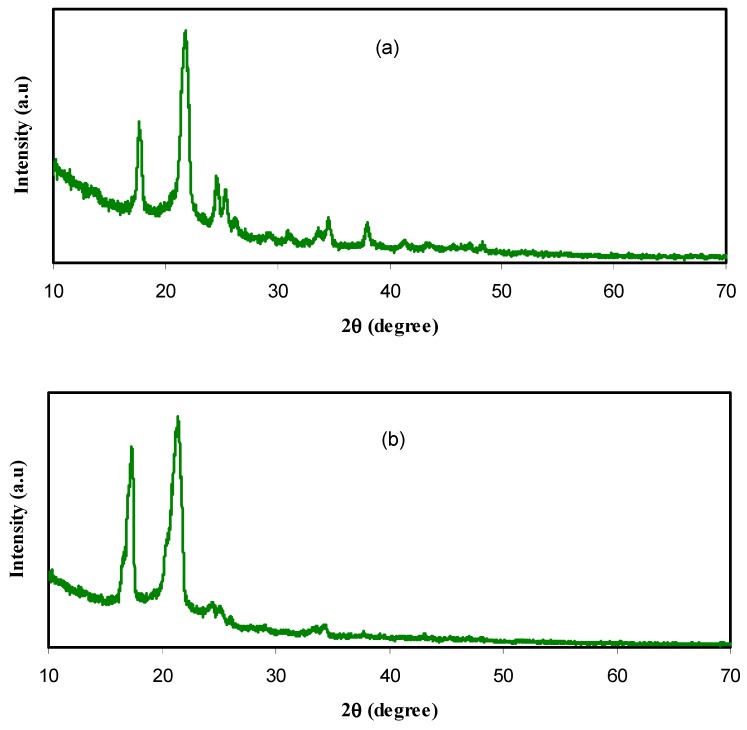

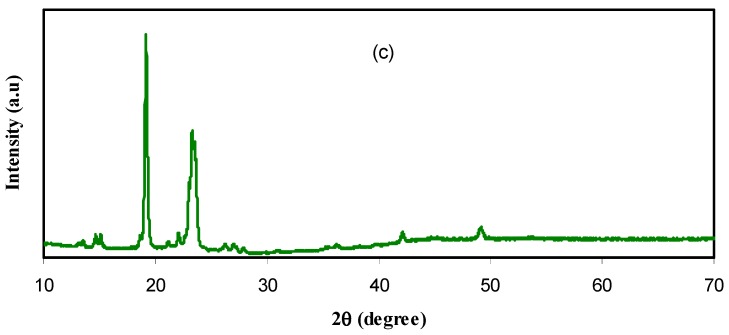
Powder X-ray diffraction (XRD) patterns for the (**a**) pure PEO (PEPN0), (**b**) PEO:CNDs (PEPN1), and (**c**) PEO:CNDs:Ag (PEPN2) samples.

A great deal of work in the field of polymer research has been carried out on PEO. This great scientific interest has been triggered not only by the prosperous dynamical, structural, and crystallization behaviors, but also by its potential use in various solid-state device applications, such as flexible electrochromic displays and rechargeable lithium batteries. Pure PEO polymer has been characterized to be in the class of semicrystalline polymers, as previously mentioned [34]. This has been confirmed by XRD analyses. In this work, XRD patterns for our pure PEO and PEO-based polymer nanocomposite samples were studied as shown in Figure 1. Notable high intensity diffraction peaks for pure PEO sample [(see Figure 1a) were found to be at 2θ = 17.66° and 2θ =21.73°, which results from the order of polyether side chains and strong intermolecular interaction connecting PEO chains through hydrogen bonding [35,36]. This finding is in agreement with the previous data obtained by Huang and Chen [37], in which the crystalline peaks were occurred at these specified 2θ angles for the pure PEO sample. It is clear from Figure 1b that the peak positions for the PEO incorporated with CNDs are changed and several peaks at higher 2θ region are almost disappeared. This indicates that the addition of CNDs to the PEO-based polymer causes a decrease in the degree of crystallinity of the PEO, which originates from the order of polyether side chains [38]. Moreover, it is interesting to note that the main peaks of PEO are further shifted to higher 2θ region as 10 mL of Ag NPs is added to the PEO:CNDs system, as shown in Figure 1c. Upon the addition of Ag NPs to the PEO:CNDs system, the main peaks were shifted from 2θ = 17.29° and 21.38° to 19.15° and 23.25°. Therefore, Ag NPS greatly affect the crystalline structure of PEO compared to CNDs particles.

### 3.2. Morphology Study

**Figure 2:**
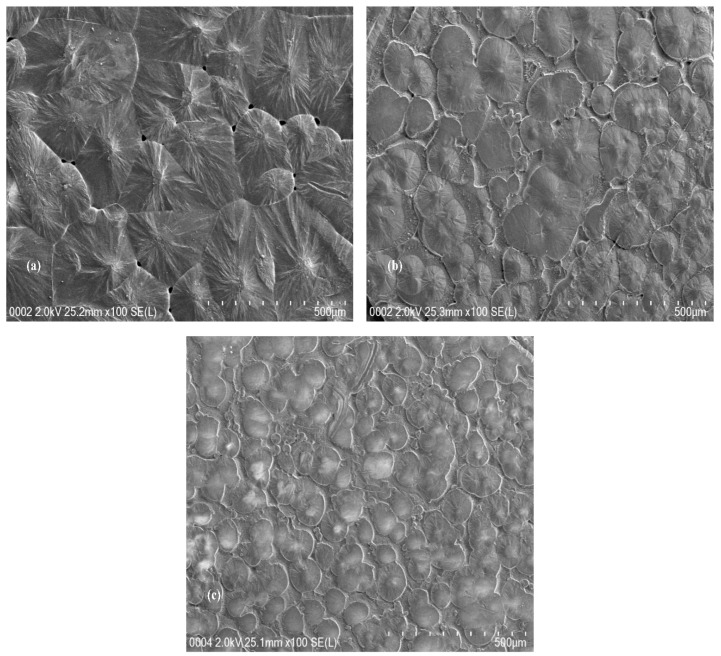
FESEM images of the (**a**) pure PEO, (**b**) PEO:CNDs, and (**c**) PEO:CNDs:Ag samples.

Study of the surface morphology can be an important tool to understand the structural changes of the samples. Earlier studies noticed that the morphological aspects must be taken into consideration in order to get more insights about the changes taken place within the polymer structure [2,35,39,40,41]. As it is well-known, polymer materials are classified into two categories, crystalline and amorphous polymers. Crystalline polymers are compact crystal assembly of stereo-regular chains, whereas, amorphous polymers exhibit rubbery or glassy behavior [42]. In our previous works, SEM technique was used to understand the compatibility of salts with polar polymers [43]. SEM technique has been proven to be an effective tool in studying the leakage of nanoparticles to the surface of polymers [12,44,45]. Figure 2 shows the morphological appearance of the pure PEO and PEO nanocomposite samples under the scanning electron microscope. It appears that the size of spherulites of crystalline regions of the PEO films become smaller upon the incorporation of CNDs particles (Compare Figure 2a,b). However, it is important to notice from Figure 1c that on the addition of Ag NPs to the PEO:CNDs system, the size of the spherulites is further reduced. This indicates that the percentage of amorphous portion in the matrix of PEO nanocomposites has been increased, owing to the distribution of CNDs and Ag NPs. PEO electrolytes are composed of variety structures, such as semicrystalline phase, amorphous phase and crystalline–amorphous interphase [34]. Typically, parts of a chain participate in the crystal phase, whereas the other parts remain in amorphous at the interface. This is intrinsically based on the topological connections [42]. The spherulitic patterns might yield useful tips for interpreting the effects of CNDs and Ag NPs on the PEO crystalline domain [46]. Various methods, such as plasticizer, blending, and filler incorporation, were introduced in literatures to reduce the crystalline phase in PEO host polymer [14,15,34,35,47]. Bandara et al., [47], noted that the spherulites in PEO incorporated with a tetrapropyl ammonium iodide system integrated with alumina have been increased. They observed that more surface area is covered by spherulites in sample include alumina compared to nonintegrated PEO:tetrapropyl ammonium iodide system. While, Koduru et al. [34] did not observe distinguishable spherulites through the SEM investigation in their study. When crystallization occurs in crystalline polymer, ordered structures are hierarchically developed on different length scales. A polymer chain usually folds back and forth, to form a folded chain lamellar crystal with a definite period over molecular dimension. This chain-folded lamellar structure has free growth on two lateral dimensions. On the other hand, it is limited in the chain extension direction, where most defects are centralized in the folding surfaces [42]. In the present work, the crystallite size of spherulites was found to drastically reduce upon addition of CNDs and Ag NPs. It is clear from Figure 2a that spherulites are connected with each other and it is difficult to distinguish their boundaries. However, for the PEO incorporated with CNDs, the boundaries of spherulites have become more evident and their sizes have reduced. Obviously the samples containing Ag NPs exhibit spherulites with smaller sizes compared to pure PEO and PEO:CND samples. The shifting of XRD peaks and disappearing of some peaks are therefore related to these changes occurring in the structure of PEO as observed by FESEM technique.

### 3.3. Optical Properties

#### 3.3.1. Absorption Study

**Figure 3:**
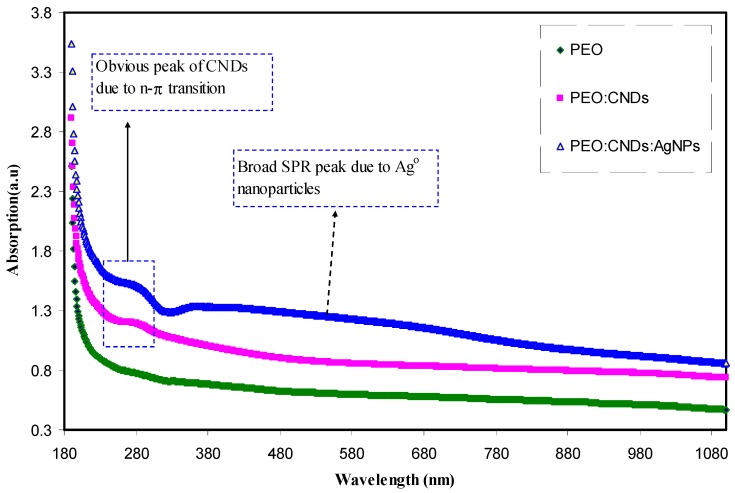
The UV–Vis absorption spectra of the pure PEO, PEO:CNDs, and PEO:CNDs:Ag composite samples.

**Figure 4:**
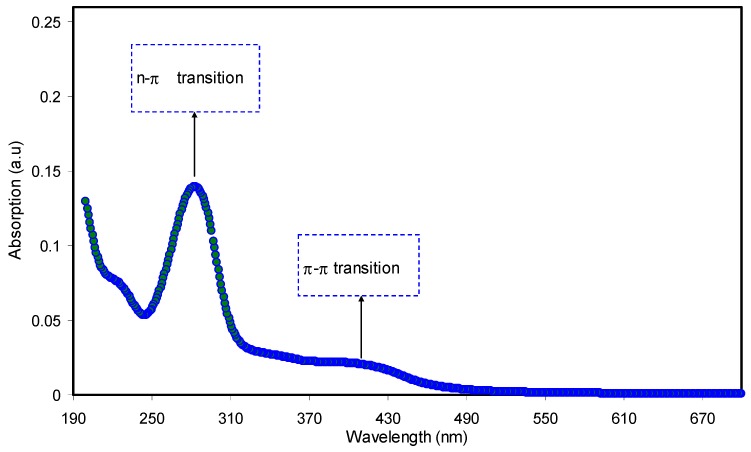
The UV–Vis absorption spectra for pure CNDs solution.

**Figure 5:**
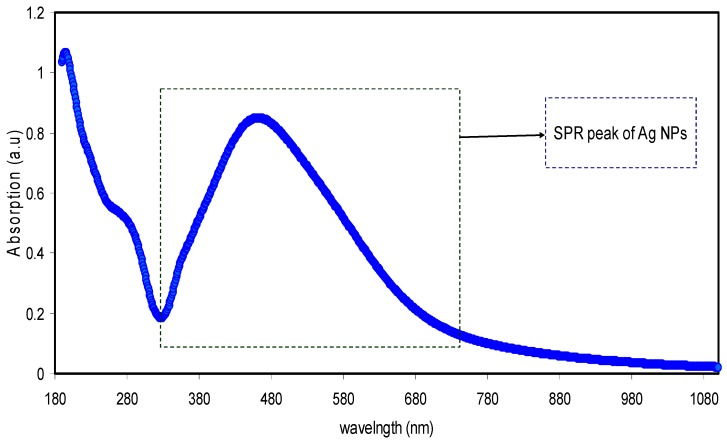
The UV–Vis absorption spectra for pure Ag NPs.

Figure 3 shows the UV–Vis absorption spectra of the pure PEO and PEO composite samples. It is obvious from the figure that, with incorporating the CNDs and Ag NPs to the PEO matrix, the absorption increases and shifts to higher wavelengths. The peak appeared at around 284 nm for PEO:CNDs and PEO:CNDs:Ag films can be ascribed to the n-to-π transition of CNDs particles [48,49,50]. To confirm that this peak is due to CNDs particles, the UV–Vis spectrum of pure CNDs suspended particles was also performed as shown in Figure 4. Here, two peaks can be seen clearly at 280 nm and 430 nm, which can be ascribed to the n–π* and π–π* transitions, respectively [2,48,49]. The absorption of light in the UV–Visible wavelength range results in electronic transitions between σ-, π-, and n-orbitals, from low-energy ground state to high-energy excited state, as described by molecular orbital theory. Consequently, the allowed transitions between these orbital electrons are σ-to-σ*, n-to-π*, and π-to-π* transitions. It is important here to mention that most of the optical transitions that are resulted from the impurities are occurred in the range of visible wavelength. Therefore, the production of defects are color centered [51]. The integration of CNDs into polymer matrices, for the applications of photonic and optoelectronic devices, is reported to be still under deep consideration [52]. Organic–inorganic composites have been widely investigated as a promising material for a new generation of optical, nonlinear optical and electronic devices as well as biological labels [53]. In contrast to the pure PEO and PEO:CNDs samples, from Figure 3, a wide absorption peak can obviously be seen for the PEO:CNDs sample incorporated with 10 mL of suspended Ag NPs. This is recognized as the silver NPs surface plasmon resonance (SPR) peak. The SPR phenomenon is not only important for a strong absorption band (usually centered in the visible spectrum), but also for the high third-order nonlinear optical susceptibility, which often originates from the large enhancement of the local electric field near the nanoclusters. Therefore, these materials are good candidates for the application of optoelectronic device, such as ultrafast optical switch [54]. The SPR peak of metal NPs are currently being used for a range of applications, such as molecular sensing and tagging, near-field optical microscopy, focusing of light and subwavelength photonic devices [55]. After all, surface plasmon is a type of free-charge oscillation taking place at the metal-dielectric interface. The surface plasmons (SPs) are generally excited through an incident light beam on the surface [56]. The presence of SPR peak in the absorption spectra is the primary signature of metal NPs formation, since SPR peak only takes place in the metal NPs [57]. Recently, many applications based on SPR of Ag NPs have been presented, in particular for biosensing, plasmon circuitry, and surface-enhanced Raman scattering [58]. Compared to our previous work [33], the SPR peaks of Ag NPs are significantly broadened. Figure 5 shows the UV–Vis spectra for the pure Ag NPs. It is obvious from the figure, the pure Ag NPs exhibits distinguishable SPR peak. While this peak is greatly broadened in PEO:CNDs system. This can be ascribed to the effect of CNDs filler. It is well stated that the large containing oxygen/hydrogen species, such as –OH and –COOH, on the surfaces of CNDs are adequate to enhance the hydrogen bonding [59,60]. It is known that fillers have weak possibility to interact with the polymer functional groups, when hydrogen bonding enhanced among the polymer chains [41,44,45]. The host polymer, therefore, may weakly influence the Ag NPs. Consequently, the SPR peak broadening possibly relates to the increase of hydrogen bonding by CNDs filler.

“Optical property” means the response of a material to the application of electromagnetic radiation, particularly, to visible light. It is sometimes more convenient to consider electromagnetic radiation from point of view of quantum mechanics, in which radiation can be treated as packets of energy called photons, rather than waves. The energy *E* of a photon is considered to be quantized and defined by the following relationship.

*E* = *hυ* = *hc*/*λ* (1)

where, *h* is Planck’s universal constant (6.63 × 10^−34^ J-s), *c* is the speed of light in vacuum (2.997 × 10^8^ m/s), and *λ* is the photon wavelength. It is well-known fact that a light wave undergoes attenuation or losses with distance as it propagates through a material. The absorption coefficient, which is described as the fractional decrease in intensity per unit distance, is defined as [61]

*α* = −1/I * dI/*dx* = (2.303/*d*) × *A* (2)

where, *I* is the intensity.

**Figure 6:**
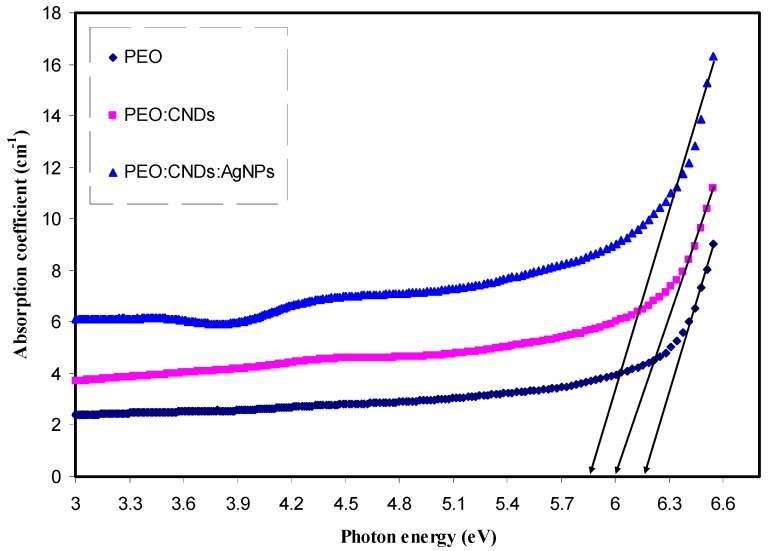
The variation of absorption coefficient as a function of photon energy for the pure PEO and PEO composite samples at room temperature.

The optical properties of solid-state materials offer a valuable resource for studying energy band structure, lattice vibrations, impurity levels, localized defects, excitons, and certain magnetic excitations. A photon can excite an electron from a filled state in the valence band to an empty state in the conduction band. This process is known as an interband transition and is purely quantum mechanical in nature [62]. In this process, a photon is absorbed to produce an excited electronic state and leaving behind an “electron hole”. In later section, more insights about the electronic transition and band gap study can be grasped. The variation of the absorption coefficients as a function of photon energy for the pure PEO and PEO composite samples were obtained from absorbance measurements at room temperature as shown in Figure 6. The absorption edge values for each sample are tabulated in Table 1. It is clear from the results that the PEO absorption edge has shifted toward lower photon energy with the addition of CNDs and Ag NPs. Such shifting towards the lower photon energy indicates that the optical band gap energy for the doped samples has been decreased [63].

#### 3.3.2. Photoluminescence (PL) Study

**Figure 7:**
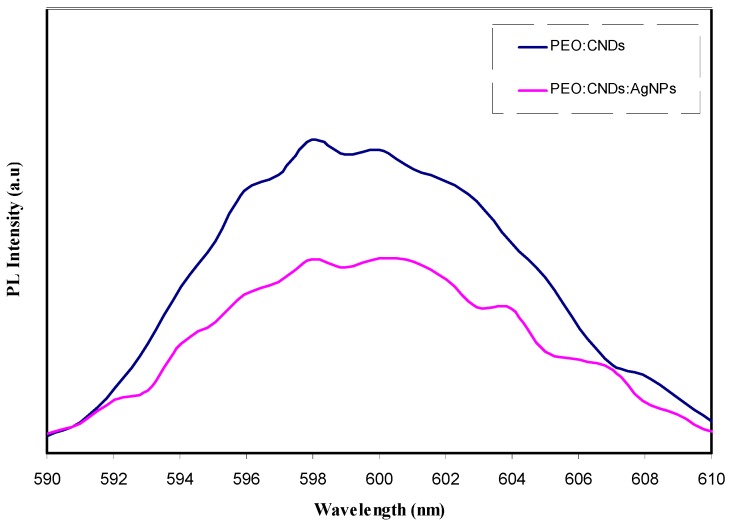
The photoluminescence (PL) spectra at excitation wavelength of 300 nm for the PEO:CNDs (PEPN1) and PEO:CNDs:Ag (PEPN2) nanocomposites.

Figure 7 shows the photoluminescence (PL) spectra at excitation wavelength of 300 nm for PEO:CNDs and PEO:CNDs:Ag composites samples. It can be seen that PL emission of PEO:CNDs composites are more intense than that of PEO:CNDs incorporated with Ag NPs. It is obvious that the PL spectrum of PEO:CNDs:Ag system was decreased. The prepared Ag NPs display photoluminescence as a result of electron excitation from occupied d-bands into unoccupied states above the Fermi level. Subsequent relaxation through electron–phonon- and hole–phonon-scattering processes result in an energy loss and, consequently, photoluminescent radiative recombination of an electron from an occupied sp band with the hole occurs [64]. The characteristic PL band in both PEO:CNDs and PEO:CNDs:Ag composites was observed to be broad. Other researchers have also demonstrated a broad-band emission characteristic for dyes doped with silver NPs at ~520–600 nm [65]. Yeshchenko et al., [66], noticed that the PL total intensity can be greatly decreased by increasing temperature, indicating a strong dependence of the PL quantum yield of Ag NPs on temperature. On the other hand, in recent years, the enhancement of PL characteristics has been studied for dye and complex molecules in accordance with surface plasmon excitation based on metal nanoparticles [65]. There is a growing literature on the study of optical and electronic properties of silver nanostructures, in particular of Ag NPs. The luminescence of silver and noble metals is usually based on transitions of electrons between the upper d-band and conduction sp-band. The luminescence of silver metal can be induced by illuminating the metal (or film) surface with a beam of photon, electron or laser beam. The position of the luminescence emission peaks has been reported to be distributed over a wide range of wavelength, from 320 to 520 nm. The plasmon absorption peak at around 400 nm and above is known to be characteristic of nano-sized silver particles [67]. The visible luminescence from Ag NPs has been reported earlier and ascribed to the excitation of electrons from occupied d-bands into unoccupied states above the Fermi level [68]. Based on SPR excitation, the decrease of PL intensity can be interpreted. It is well reported that the enhancement of the PL is associated with the SPR excitation [29]. Therefore, the decrease in SPR peak intensity gives rise to weak PL spectra. The results of PL study are completely in agreement with the results of UV–Vis absorption study. From the UV–Vis spectra of Figure 3, it was observed that the surface plasmonic resonance of Ag NPs is well broadened. It can be established that this broadening is responsible for the reduction of intensity of PL spectra for the sample incorporated with Ag NPs.

#### 3.3.3. Refractive Index Study

When a light ray passes from one medium (e.g., air) into another (e.g., solid), there are several things that can take place. Some of the light ray may be reflected, some absorbed and some transmitted through the medium. The intensity of the incident beam to the surface of second medium ($I_{o}$) must be equal to the sum of the intensities of the reflected, absorbed and transmitted beams, denoted as $I_{R}$, $I_{A}$, and $I_{T}$, respectively, or

(3) $$I_{o}=I_{R}+\;I_{A}+I_{T}$$

Radiation intensity measured in watts per square meter refers to the energy transmitted per unit time per unit area that is perpendicular to the direction of propagation. Therefore, by dividing Equation (3) by $I_{o}$, one can obtain the following formula.

*R* + *A* + *T* = 1 (4)

where, *R*, *A*, and *T* represent, respectively, the reflectivity ($I_{R}/I_{o}$), absorptivity ($I_{A}/I_{o}$), and transmissivity $I_{T}/I_{o}$, or the fractions of incident light that are reflected, absorbed, and transmitted. Due to that the entire incident light is either reflected, absorbed, or transmitted, their sum must equal one [69]. The optical refractive index (*n*) of the medium, which measures the reduction rate of the light speed within the medium, is one of the important parameters that can be calculated from *R* and extinction (*K*) of the sample by using the following formula [2].

(5) $$\;n=\left[\frac{\left(1+R\right)}{\left(1-R\right)}\right]+\sqrt{\frac{4\times R}{{\left(1-R\right)}^{2}}}-K^{2}$$

where, K is directly proportional to both absorption coefficient (α) and wavelength (λ) and inversely proportional to the sample thickness (t), through K=αλ/4πt.

**Figure 8:**
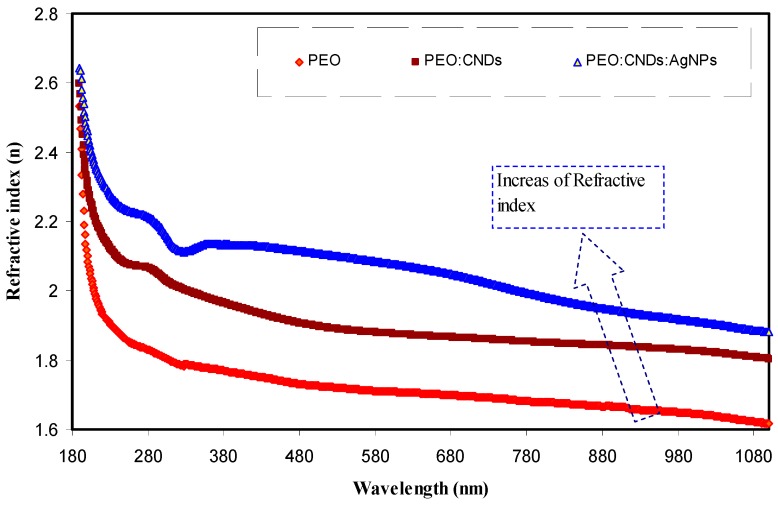
The refractive index spectra of the pure PEO (PEPN0), PEO:CNDs (PEPN1), and PEO:CNDs:Ag (PEPN2) composite samples.

From Equations (3) and (4), one can clearly compute *R* from the values of *A* and *T*, i.e., using R=1−(A+T), whereas the value of *T* can be obtained by use of Beer’s law (i.e., $T=10^{-A}$). Figure 8 shows the refractive index spectra of the pure PEO, PEO:CNDs, and PEO:CNDs:Ag samples. It is clear that, with increasing CNDs and Ag NPs, the refractive index has been increased. Recently, it is noted that the necessity for high optical refractive index materials in device applications is increasing, such as in applications of filters, ophthalmic lenses, highly reflective and antireflective coatings, optical adhesives and advanced optoelectric fabrications [70]. Jin et al., [71], reported that the refractive index of PMMA can be increased from 1.49 to 1.839 by adding 20 wt % of TiO_2_, which is lower than that obtained in our work for PEO incorporated with CNDs and Ag NPs.

#### 3.3.4. Band Gap Study

**Figure 9:**
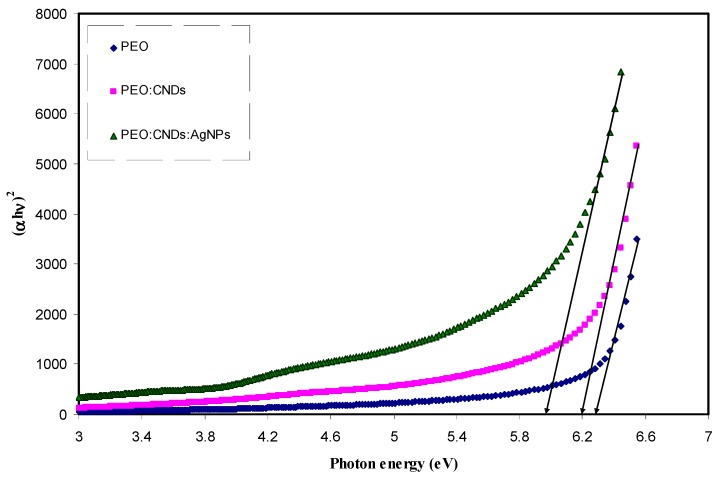
Plot of *(αhυ)*^2^ vs. photon energy for pure PEO (PEPN0), PEO:CNDs (PEPN1), and PEO:CNDs:Ag (PEPN2) composite samples.

**Figure 10:**
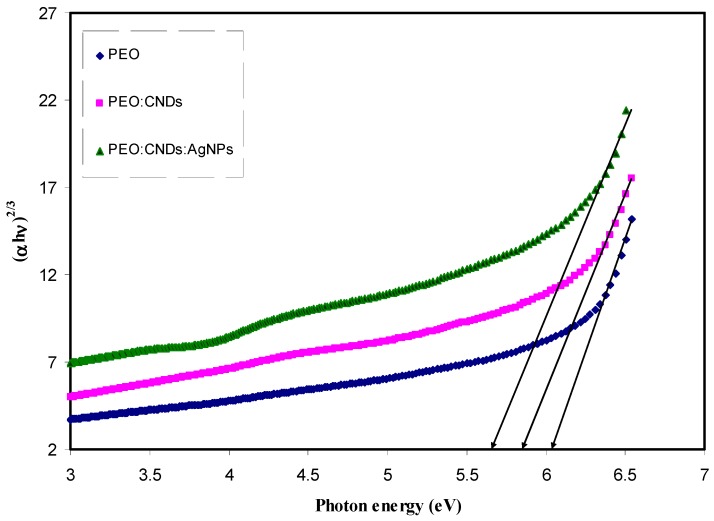
Plot of *(αhυ)*^2/3^ vs. photon energy for pure PEO (PEPN0), PEO:CNDs (PEPN1), and PEO:CNDs:Ag (PEPN2) composite samples.

**Figure 11:**
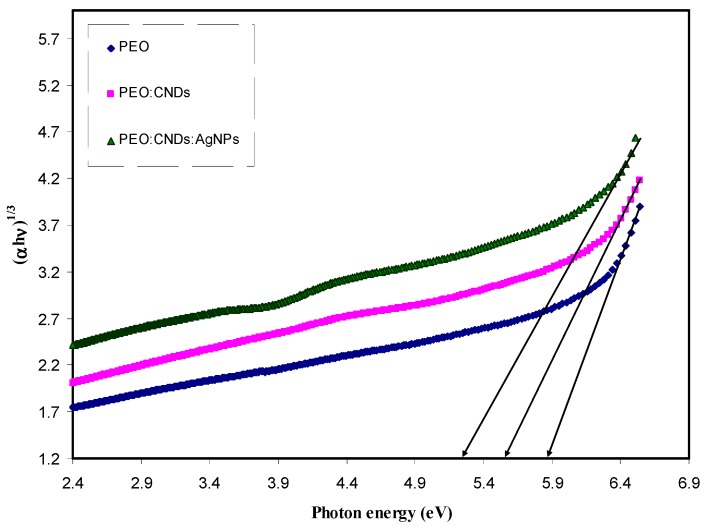
Plot of *(αhυ)*^1/3^ vs. photon energy for pure PEO (PEPN0), PEO:CNDs (PEPN1), and PEO:CNDs:Ag (PEPN2) composite samples.

**Figure 12:**
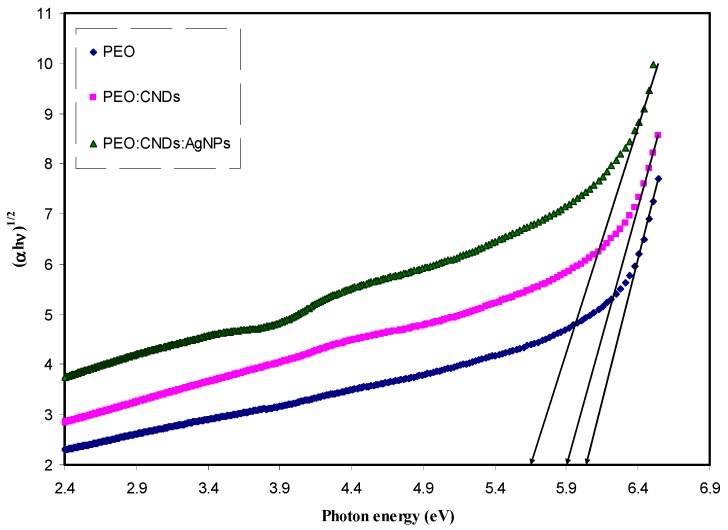
Plot of *(αhυ)*^1/2^ vs. photon energy for pure PEO (PEPN0), PEO:CNDs (PEPN1), and PEO:CNDs:Ag (PEPN2) composite samples.

In insulators, the energy band gap is relatively large enough such that at ambient temperature, essentially no free carriers can thermally excite across the energy band gap. This implies that there is no carrier absorption. Therefore, interband transitions only appear to be important at relatively high photon energies (above the visible). This explains why many insulator materials are optically transparent. However, in metals, free electron absorption is extremely significant and occurs at energy of about 10 eV far away from the ultraviolet region [62]. The evaluation of optical band gap from absorption coefficient data in response to wavelength is actually found to be in dependence with Tauc’s relation [72,73]:

(*αhv*)^1/*n*^ = *B*(*hv* − *E_g_*) (6)

where, *hυ* is the photon energy (eV), *α* is the absorption coefficient, *B* refers to the band form parameter, *E_g_* denotes the optical band gap, and *n* = 1/2 for direct band gap transition; *n* = 2 for indirect band gap transition. Also *n* takes the values equal to 3/2 and 3 for direct forbidden and indirect forbidden transitions, respectively [2]. Figure 9, Figure 10, Figure 11 and Figure 12 show the four plots of ${\left(\alpha h\upsilon \right)}^{1/n}$ as a function of hυ for *n* = 2, 1/2, 2/3, and 1/3, respectively. The optical band gap of the samples was determined by extrapolating of linear part of the curves to the zero absorption value. The estimated values are listed in Table 2. It is obvious from all value of n that the band gap values for the pure PEO have been decreased with the incorporation of CNDs and Ag NPs. Mohan et al., [15], in which the optical properties of PEO:NaFeF4-based solid polymer electrolytes were studied, also plotted the α and ${\left(\alpha h\upsilon \right)}^{2}$ as a function of Photon energy (*hυ*) to observe the nature and width of the band gap. They observed that the absorption edge of the pure PEO film was around 4.62 eV, whereas, with the NaFeF4 doped, the value of absorption edge had shifted toward lower photon energies. However, in another study, Mohan et al. found that the optical band gap was not affected greatly by incorporating the PEO with different amount of NaLaF4 salt [74]. On the other hand Ibrahim et al., [75], studied the optical properties of plasticized nanocomposite polymer electrolytes based on PEO. They observed that the optical band gap of pure PEO does not have a good response to the addition of LiPF6, plasticizer (EC), and amorphous carbon nanotubes. In this work, our estimated band gap value for the pure PEO sample is found to be in a good agreement with the value obtained by Ibrahim et al. I From the above discussion, it appears that it is difficult to determine the types of electronic transition from the Tauc’s relation in considering four plots for all n values. Therefore, in order to specify the exact type of electronic transition and band gap, the optical dielectric loss parameter must be studied. More insights about the optical dielectric loss parameter can be seen in later section.

In semiconducting/conducting polymers, electronic transition and charge transfer complexes are poorly understood. The transitions take place by providing required energy and momentum from the incident photon and phonon, respectively [76]. Earlier studies established that, to determine the optical band gap and electronic transition types, optical dielectric loss and Tauc’s model can be used, respectively [2,7,11,12,33]. This is related to the fact that the optical dielectric function hardly depends on the material’s band structure. Therefore, a more precise description of this correlation would require a proper consideration of quantum physics of complex dielectric function. This is due to the fact that the complex dielectric function ($\varepsilon^{*}$) explains the electronic response of electron density of a material to an applied electromagnetic wave [77]. However, it is difficult to predict, from the Tauc’s equation, whether the band structure will be a direct or an indirect type, as discussed earlier [78]. A real transition between the occupied and unoccupied states (i.e., $\Psi_{k}^{v}$ and $\Psi_{k}^{c}$ wave functions, respectively) can be represented by the imaginary part (ε_2_) of complex dielectric function (ε) using [79]

(7) $$\varepsilon_{2}=\frac{4\pi^{2}e^{2}}{m^{2}\omega^{2}V}\underset{v,c,k}{\sum }\left|\langle \Psi_{k}^{v}|\vec{p_{i}}{|\Psi }_{k}^{c}\rangle \right|\delta \left(E_{\Psi_{k}^{c}}-E_{\Psi_{k}^{v}}-\hbar \omega \right)$$

**Figure 13:**
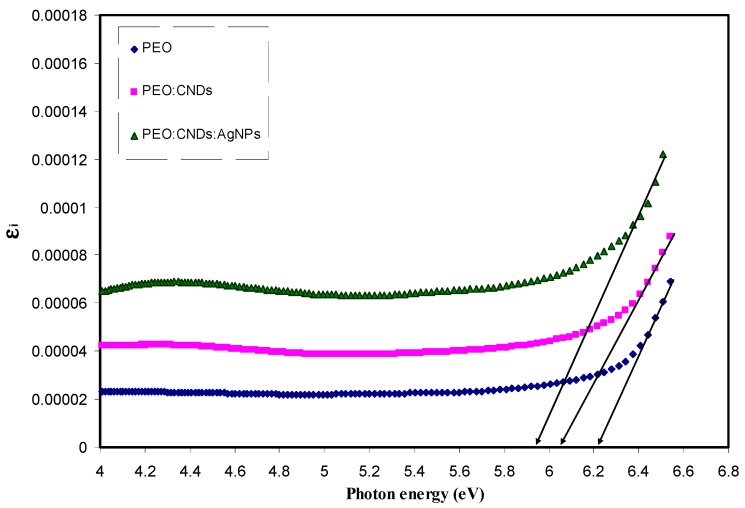
Optical dielectric loss spectra of pure PEO (PEPN0), PEO:CNDs (PEPN1), and PEO:CNDs:Ag (PEPN2) composite samples.

**Figure 14:**
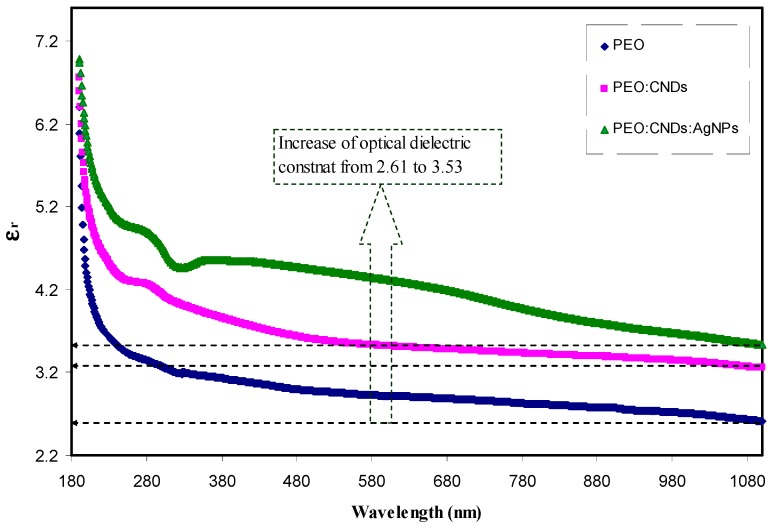
Optical dielectric constant spectra of pure PEO (PEPN0), PEO:CNDs (PEPN1), and PEO:CNDs:Ag (PEPN2) composite samples.

Therefore, it is clear from Equation (7) that the optical dielectric loss, i.e., $\varepsilon_{2}$, is correlated with the band structure ($E_{\Psi_{k}^{c}}-E_{\Psi_{k}^{v}}$) of the material. Through using a few simple equations, the complex dielectric function, which is related to other measurable optical parameters, such as refractive index and extinction coefficient, can be calculated. Figure 13 shows the optical dielectric loss versus the applied photon energy for all the samples. For all the samples, clear linear parts can be noted. Previous studies confirmed that the linear parts in $\varepsilon_{2}$ spectra are closely associated with the inter-band transitions [2,7,11,12,33]. Therefore, the intercept of linear parts (see Figure 13) with the photon energy axis can be regarded as the real band gap. Then, by comparing the plots obtained from Tauc’s equation (see Figure 9, Figure 10, Figure 11 and Figure 12) to Figure 13 of optical dielectric loss, the types of electronic transition can be identified. It is possible to conclude that the type of electronic transition in pure PEO and PEO:CND:Ag samples are direct allowed, while for the PEO:CNDs sample, the transition is mainly indirect allowed. Therefore, optical dielectric function can be a useful tool to study the band gap structure for solid materials. The decrease of band gap (see Table 2) is related to the increase density of states upon addition of CNDs and Ag NPs to the PEO host polymer. This can be more understood through the study of optical dielectric constant. To a large extent, there is evidence that optical dielectric constant ($\varepsilon_{1}$) parameter is related to the density of states, which directly related to the localized electronic states in the forbidden gap of materials [2,6]:

(8) $$\varepsilon_{1}=n^{2}-k^{2}=\varepsilon_{\infty }-\frac{e^{2}}{4\pi C^{2}\varepsilon_{o}}\frac{N}{m^{*}}\lambda^{2}$$

where $\varepsilon_{\infty }$ and $\varepsilon_{o}$ refer to the dielectric constant at infinite wavelength and free space dielectric constant, respectively. k is the extinction coefficient, N/m* is the ratio of localized electronic state density to the effective mass, and the other symbols have their usual meanings. Figure 14 shows the variation of $\varepsilon_{1}$ with wavelength at different CNDs concentrations. The value of $\varepsilon_{1}$ is found to have a direct proportionality with the concentration of CND and Ag. An increase in $\varepsilon_{1}$ value from 2.6 to 3.5 is found and can be attributed to the increase of density of states in the consequence of direct correlation of $\varepsilon_{1}$ parameter to the density of states within the forbidden gap of the solid polymer films [80].

## 4. Conclusions

In summary, we have studied the reduction in crystallite size of spherulites in PEO-based polymer composite. The broadening of SPR peaks of Ag NPs in PEO-based polymer nanocomposites mediated by CND particles also is another conclusion in this work. Green approaches have been used to synthesis CNDs and Ag NPs. Solution cast techniques were employed to prepare PEO-based polymer nanocomposites. Various techniques, such as XRD, FESEM, UV–Vis, and PL, have been carried out for the characterization of the samples. The XRD results indicated that the amorphous phase of PEO polymer matrix increased upon addition of CND particles. The position of XRD peaks were further changed with the adding of Ag NPs to the PEO:CNDs composites. The morphological appearance of the samples was also studied using FESEM. The size of spherulites drastically reduced upon addition of CND and Ag NPs to the PEO host polymer. The UV–Vis absorption spectra were obtained for all the samples. The results showed that the absorption spectra of PEO improve and shift to higher wavelengths as CNDs and Ag NPs add to the PEO host polymer. The SPR peaks, in the PEO:CNDs:Ag system, were found to become broader due to Ag NPs. The absorption edge value of PEO was shifted to lower photon energies when CNDs and Ag NPs are added. The PL spectra of the PEO:CNDs system was found to be more intense than that of PEO:CNDs:Ag system. Through using Tauc’s model and optical dielectric loss parameter, the optical band gap of the samples was studied in detail. Sufficient experimental data and quantum models were presented to confirm that optical dielectric loss parameter is effective to study the energy band gap. The obtained band gap values from Tauc’s model were compared to those obtained from the optical dielectric loss parameter to specify the type of electronic transition.

## Figures and Tables

**Table 1:** Absorption edge values for pure PEO (PEPN0), PEO:CNDs (PEPN1), and PEO:CNDs:Ag (PEPN2) composite samples.

Sample Code	*Absorption Edge (eV)*
PEPN 0	6.27
PEPN 1	6
PEPN2	5.84

**Table 2:** Estimated optical band gap from Tauc’s method [(αhυ)^1/n^ vs. hυ] and optical dielectric loss plot.

Sample Code	*E_g_* for *n* = 1/2	*E_g_* for *n* = 2	*E_g_* for *n* = 3	*E_g_* for *n* = 3/2	*E_g_* from *ε_i_* Plot
PEPN 0	6.26	6.07	5.83	6.07	6.22
PEPN 1	6.2	5.9	5.58	5.84	6.05
PEPN2	6	5.65	5.35	5.7	5.95
